# Transforming Eye-Care Diagnostics Through Artificial Intelligence, Biometric Evaluation, and Tele-Optometry

**DOI:** 10.7759/cureus.107620

**Published:** 2026-04-23

**Authors:** Sushil Kumar Kar, Kedar Nemivant, Urvashi Krunalkumar Sharma, Priyanshi Priya, Sadaf Abbasi, Niraj Kumar Yadav

**Affiliations:** 1 Department of Ophthalmology, Hi-Tech Medical College & Hospital, Rourkela, IND; 2 Department of Ophthalmology, All India Institute of Medical Sciences, Rajkot, Rajkot, IND; 3 Department of Microbiology, Dr N.D. Desai Faculty of Medical Science and Research, Dharmsinh Desai University, Nadiad, IND; 4 Department of Ophthalmology, Dr. KNS Memorial Institute of Medical Sciences, Barabanki, IND; 5 Department of Ophthalmology, King George's Medical University, Lucknow, IND

**Keywords:** artificial intelligence, biometric evaluation, ophthalmic diagnostics, tele-optometry, vision screening

## Abstract

Visual impairment remains a major global health concern associated with disorders such as diabetic retinopathy, glaucoma, cataract, and age-related macular degeneration. Early detection of ocular abnormalities remains essential for preventing irreversible visual loss. Conventional diagnostic methods rely on clinical examination and specialised imaging, yet limitations in accessibility, diagnostic variability, and delayed detection continue to affect effective eye-care delivery in many settings. Advances in digital technologies have introduced innovative diagnostic approaches integrating artificial intelligence (AI), ocular biometric evaluation, and tele-optometry to improve efficiency and reach of ophthalmic care. The objective of this review involves a comprehensive examination of recent developments in digital ophthalmic diagnostics, focusing on AI-based image analysis, biometric measurement technologies, and tele-optometry platforms. Relevant literature addressing AI applications, ocular biometric assessment, and remote ophthalmic screening systems was evaluated to synthesise current knowledge on diagnostic capability and clinical relevance. Current evidence indicates that AI algorithms enable automated interpretation of retinal images and facilitate early identification of ocular diseases. Biometric evaluation provides precise anatomical measurements that support refractive assessment and surgical planning. Tele-optometry systems expand diagnostic access through remote imaging and consultation services, improving screening coverage in underserved populations. Integration of AI, biometric technologies, and tele-optometry demonstrates strong potential to enhance disease detection, strengthen clinical decision support, and expand accessibility of ophthalmic diagnostic services.

## Introduction and background

Visual impairment is a global health problem with significant clinical, economic, and social impacts on healthcare systems worldwide [1]. Among the leading causes of preventable vision loss are progressive eye diseases such as diabetic retinopathy, glaucoma, cataract, and age-related macular degeneration, with diabetic retinopathy being a major contributor globally [2]. The increasing prevalence of these conditions is driven by ageing populations and rising metabolic disorders, reinforcing the need for effective screening and diagnostic strategies in ophthalmic care [2]. Early identification of pathological alterations in ocular tissues remains critical for preventing irreversible vision loss and preserving visual function [3]. Traditional diagnostic pathways in ophthalmology are primarily clinician-dependent, relying on imaging modalities such as slit-lamp biomicroscopy, fundus examination, optical coherence tomography, and visual field analysis [4]. While these approaches are effective in well-equipped settings, they are constrained by limited accessibility, inter-observer variability, and delayed diagnosis in community and primary care settings [5]. Geographic disparities in specialist availability and the operational complexity of population-based screening programs further restrict the timely detection of retinal and optic nerve diseases [6].

Building on these limitations, advances in medical imaging and computational sciences have driven a transition toward digital ophthalmology, enabling data-driven, scalable, and reproducible diagnostic approaches. Artificial intelligence (AI) technologies have demonstrated strong potential in automated analysis of retinal images and optical coherence tomography data, with diagnostic accuracy comparable to expert evaluation in selected screening contexts [7]. Recent advances include the application of machine learning and deep learning models, particularly convolutional neural networks (CNNs), which enable automated feature extraction, pattern recognition, and classification of retinal and optic nerve pathologies [8]. These developments support improved efficiency, objectivity, and scalability in ophthalmic diagnostics [9]. Despite these advances, significant variability persists in diagnostic performance across populations and clinical environments. Complementing AI-based approaches, ocular biometric evaluation provides a quantitative assessment of anatomical parameters, such as axial length, corneal curvature, anterior chamber depth, and lens thickness, which are essential for refractive assessment, surgical planning, and disease monitoring [4].

Mobile retinal imaging systems and tele-retinal screening programs have demonstrated effectiveness in identifying retinal diseases in large populations [10,11]. Despite these advancements, several challenges remain, including limited generalisability of AI models across diverse populations, variability in imaging protocols, and lack of standardised integration frameworks within clinical workflows [12]. Addressing these limitations requires large-scale validation, interoperable data systems, and robust regulatory and clinical implementation strategies, with increasing emphasis on formal regulatory frameworks and prospective multicentre validation studies to ensure safe and generalisable deployment.

Objectives of the review

This review provides an overview of recent developments in AI, ocular biometric evaluation, and tele-optometry in ophthalmic diagnostics. The discussion highlights key advances in digital imaging technologies, computational diagnostic models, biometric measurement systems, and remote screening platforms, with emphasis on diagnostic performance, clinical applicability, and integration into ophthalmic care pathways.

Methodology

This narrative review is based on a structured literature search and targeted appraisal of published studies related to AI, ocular biometric evaluation, and tele-optometry in ophthalmic diagnostics. A comprehensive search was conducted across multiple electronic databases, including PubMed, Scopus, and Web of Science, to identify relevant literature. The search covered publications from January 2015 to March 2026 to capture recent technological advancements in digital ophthalmology. Search strategies incorporated combinations of keywords and MeSH terms, such as "artificial intelligence in ophthalmology", "ocular biometrics", "tele-optometry", "retinal imaging", and "digital ophthalmic diagnostics". Boolean operators (AND, OR) were applied to refine the search. Inclusion criteria comprised peer-reviewed articles published in English that reported on clinical applications, diagnostic performance, or technological integration of AI, biometric evaluation, or tele-optometry in ophthalmology. Both original research studies and review articles with clinically relevant findings were considered.

Exclusion criteria included non-English publications, conference abstracts without full text, editorials, letters, and studies lacking direct relevance to ophthalmic diagnostics. The screening process involved title and abstract review followed by full-text assessment to ensure relevance and eligibility. Duplicate records were removed before screening. Screening was conducted independently by two reviewers, with discrepancies resolved through discussion. Studies were selected based on their relevance to diagnostic accuracy, clinical applicability, and implementation in ophthalmic care systems. Although a structured search strategy was applied, this review remains a narrative synthesis and does not follow a formal systematic review protocol such as Preferred Reporting Items for Systematic Reviews and Meta-Analyses (PRISMA).

## Review

Improvements in digital ophthalmic diagnostic technologies

High-resolution digital imaging technologies form a core component of modern ophthalmic diagnostics, enabling detailed visualisation and quantitative analysis of ocular structures. Contemporary systems utilise advanced imaging modalities to improve precision and reproducibility in assessing retinal and optic nerve anatomy [13,14]. Techniques such as optical coherence tomography and fundus imaging allow detection of microstructural changes associated with early disease processes, supporting timely diagnosis and monitoring of disease progression [15]. Integration of imaging technologies with analytical software further enhances objective evaluation and diagnostic sensitivity in clinical practice [14].

Digital Ophthalmic Imaging

One of the primary aspects of ophthalmic diagnostics today is digital imaging technology. The optical coherence tomography is a non-invasive visualisation technique that provides cross-sectional images of the retinal layers using a low coherence interferometry technique that allows a detailed evaluation of the macular structure and morphology of the optic nerve [4]. Retinal imaging can be performed in high resolution and is able to detect microstructural abnormalities related to diabetic retinopathy, glaucoma, and macular degeneration [1]. Fundus photography generates detailed two-dimensional images of the retina and optic disc, which are useful in documenting the pathological changes and following retinal disorders throughout the lifespan [10]. The use of digital fundus imaging platforms enables the quick capture of retinal photographs, which can be used to make clinical screening programs and tele-retinal consultation services [3].

Another significant diagnostic technology that is applied in the functional assessment of visual pathways is automated visual field systems. Perimetric testing measures visual field sensitivity in various regions of the retina, and it helps in detecting the early stage of glaucomatous damage and neurological visual impairment [11]. A combination of the imaging data and the computerised analysis would further enhance diagnostic accuracy with objective evaluation of structural and functional ocular changes [12].

Transition Toward Data-Driven Diagnostics

The expansion of digital ophthalmic imaging has enabled the development of multimodal diagnostic frameworks that integrate imaging and biometric data [15]. While these systems improve analytical depth and support standardised evaluation, their effectiveness is influenced by variability in data quality, imaging protocols, and device-specific differences [16]. Table 1 summarises key digital imaging technologies used in ophthalmic diagnostics and their clinical applications.

**Table 1:** Digital Ophthalmic Imaging Technologies and Clinical Uses Compiled by the authors from multiple sources [4,10,11,13]. OCT: Optical Coherence Tomography, FP: Fundus Photography, AVF: Automated Visual Field, OCTA: Optical Coherence Tomography Angiography, WF-FP: Wide-Field Fundus Photography

Technology	Diagnostic Principle	Primary Clinical Applications	Key Advantages	Limitations	Key Supporting Study
OCT	Cross-sectional retinal imaging using low-coherence interferometry	Macular disorders, glaucoma evaluation, and retinal thickness analysis	High-resolution structural imaging, non-invasive assessment	High equipment cost, limited portability	[4]
FP	Two-dimensional retinal photography using digital cameras	Diabetic retinopathy screening, retinal documentation	Rapid image acquisition, suitable for tele-screening	Limited depth visualisation	[10]
AVF	Automated perimetric measurement of visual field sensitivity	Glaucoma detection, neurological visual defects	Functional assessment of visual pathways	Patient-dependent test reliability	[11]
OCTA	Non-invasive imaging of retinal microvasculature	Diabetic retinopathy, vascular abnormalities	Visualisation of retinal blood flow without dye injection	Motion artifacts, complex interpretation	[4]
WF-FP	Wide-field retinal imaging technology	Peripheral retinal disease detection	Expanded retinal coverage	Higher equipment complexity	[13]

AI applications in ophthalmic disease detection

AI has emerged as a transformative technology in ophthalmic diagnostics due to its ability to interpret complex medical imaging data with high computational accuracy [17]. Deep learning and machine learning algorithms, particularly CNNs, enable automated retinal image analysis, facilitating early detection of pathological changes that may not be apparent during routine clinical examination [18]. Commonly utilised architectures include ResNet, EfficientNet, VGGNet, Inception networks, and U-Net models, which are optimised for tasks such as classification, segmentation, and lesion detection [15,17]. Integration of AI with ophthalmic imaging supports rapid image processing, objective pattern recognition, and enhanced diagnostic efficiency in clinical screening settings [19]. These systems are trained on large annotated datasets, allowing identification of disease-specific morphological features and generation of predictive diagnostic outputs [20]. Widely used datasets include EyePACS, Messidor, DRIVE, STARE, and UK Biobank retinal imaging datasets, which provide diverse imaging data for model training and evaluation [18]. Model development typically involves training, validation, and test dataset partitioning, with additional techniques such as k-fold cross-validation and external validation using independent datasets to ensure robustness and generalisability [7]. Performance of AI models is commonly evaluated using metrics such as accuracy, sensitivity, specificity, area under the receiver operating characteristic curve (AUC-ROC), F1-score, and precision-recall analysis, enabling objective comparison of diagnostic performance [18].

AI in Retinal Disease Screening

Applications of AI have demonstrated significant utility in retinal disease screening through automated analysis of fundus photographs and optical coherence tomography images [21,22]. Deep learning models can detect subtle retinal abnormalities, such as microaneurysms, haemorrhages, exudates, and retinal thickening associated with diabetic retinopathy [18]. Automated systems also identify pathological features of age-related macular degeneration, including drusen formation, retinal pigment epithelium alterations, and macular structural disruption [22]. These capabilities enable large-scale, high-throughput screening and support early diagnosis and timely referral in both community-based and population-level screening programs [17,18]. High-performing models in diabetic retinopathy screening have reported AUC values exceeding 0.90 in controlled validation settings, demonstrating strong diagnostic capability [8].

AI in Glaucoma and Optic Nerve Assessment

**Figure 1:**
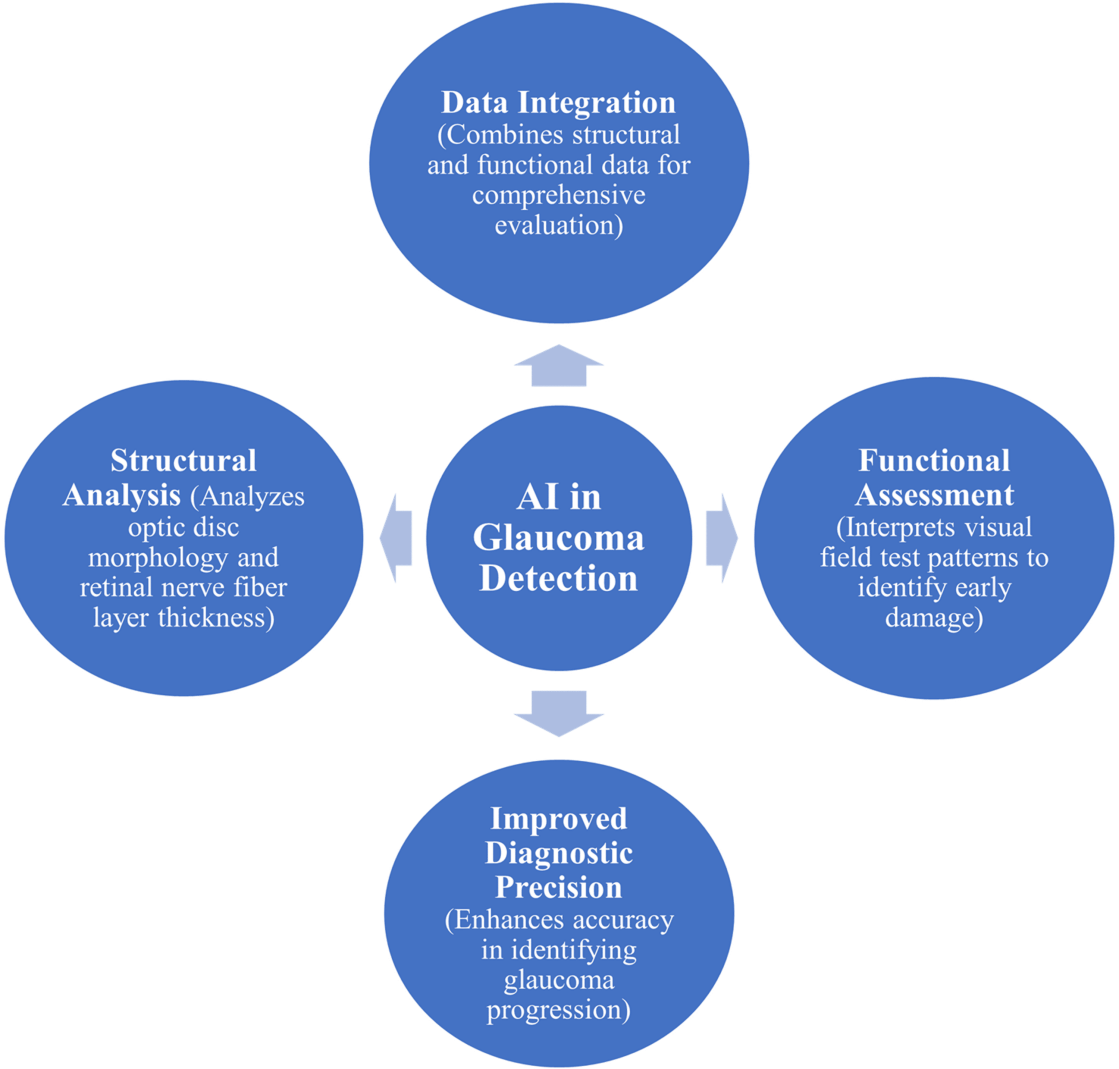
AI Framework for Glaucoma Detection Images created by authors using Microsoft PowerPoint (Microsoft® Corp., Redmond, WA).

AI techniques have also demonstrated effectiveness in detecting glaucomatous structural and functional abnormalities in the optic nerve and visual pathways [20]. Machine learning models analyse optic disc morphology, retinal nerve fibre layer thickness, and cup-to-disc ratio to identify patterns associated with glaucoma progression [21]. Architectures such as U-Net are frequently used for optic disc and retinal nerve fibre layer segmentation, while classification networks such as ResNet and EfficientNet are applied for glaucoma detection tasks. Computational analysis of visual field data further supports early detection of glaucomatous damage before clinically significant vision loss occurs [23]. Integration of structural imaging and functional assessment using AI enhances diagnostic accuracy and enables comprehensive evaluation of optic nerve health in glaucoma screening programs [20]. Nevertheless, the interpretability of these models remains limited, as many deep learning systems function as black-box algorithms. This lack of transparency may reduce clinician trust and complicate integration into routine decision-making processes. Figure 1 illustrates the AI-based framework for glaucoma detection, integrating structural, functional, and multimodal data. 

Ocular biometric evaluation in clinical eye care

Quantitative examination of the anatomical parameters is an inseparable component of ophthalmic diagnosis today through ocular biometric evaluation, which affects the visual performance and refractive state [23]. Proper scanning of eye configuration allows one to define the morphology of the eye in detail and to help in a more efficient strategy to treat eye diseases of diverse natures [24]. Current biometric systems use optical systems and ultrasound systems that can produce very precise dimensions of intraocular and corneal properties [25]. Such measurements facilitate assessment of refractive errors, tracking of ocular growth patterns, and calculation of correct intraocular lens power in cataract surgery [24].

Key Biometric Parameters

Several biometric variables offer vital data on the structure of the eyes and the visual performance. The axial length measure is used to calculate the distance between the anterior surface of the cornea and the pigment epithelium of the retina, one of the most important parameters in the calculation of refractive errors and intraocular lenses [26]. Corneal curvature refers to the curvature of the cornea and the refractive power of the lens, which determines the focusing capability of the rays of incoming light on the retina, and helps in determining visual quality [23]. The depth of the anterior chamber is an indication of the spatial separation between the back of the cornea and the front of the lens diaphragm and plays a role in the determination of anterior chamber geometry and the examination of glaucoma risks [27]. The lens thickness measurement gives data about the morphology of crystalline lenses and helps clinical evaluation of the changes in accommodation and cataract formation [28]. Despite their precision, biometric measurements may vary across devices and acquisition techniques, potentially affecting reproducibility. Such variability can introduce inconsistencies when integrating data across different clinical systems.

Clinical Utility of Biometric Measurements

Ocular biometric data are used in clinical practice throughout several ophthalmic interventions. Correct measurements of the axial length and corneal curvature also provide an accurate calculation of intraocular lens power in cataract surgery to enhance the refractive outcome after surgery [24]. The biometric analysis also helps evaluate the suitability of refractive surgery by evaluating the corneal shape, ocular size, and anatomy of the anterior segment [23]. Axial length progression monitoring aids in early identification and treatment of progressive myopia and angle-closure risk in those populations at risk [26]. Key biometric parameters and their clinical significance in ophthalmic practice are summarised in Table 2.

**Table 2:** Key Ocular Biometric Parameters and Clinical Applications Compiled by the authors from multiple sources [23,24,26-28]. AL: Axial Length, K: Keratometry, ACD: Anterior Chamber Depth, LT: Lens Thickness, WTW: White-to-White Corneal Diameter, IOL: Intraocular Lens

Parameter	Measurement Technique	Clinical Applications	Diagnostic Significance	Common Devices	Supporting Reference
AL	Optical or ultrasound biometry	Cataract surgery planning, refractive error evaluation	Determines ocular length and refractive status	Optical biometer	[26]
K	Keratometry, corneal topography	Refractive surgery assessment, IOL calculation	Indicates corneal refractive power	Keratometer	[23]
ACD	Optical biometry, anterior segment imaging	Glaucoma risk assessment, IOL positioning	Evaluates anterior segment configuration	Optical biometer	[27]
LT	Optical biometry	Cataract evaluation, accommodation analysis	Reflects lens morphology and ageing changes	Optical biometer	[28]
WTW	Corneal diameter measurement	IOL sizing, refractive surgery planning	Assesses corneal diameter	Corneal imaging device	[24]

Integration of AI with ocular imaging and biometric data

Retinal imaging, optical coherence tomography, and biometric measurements have been used to produce digital datasets that are increasingly used in modern ophthalmology to produce clinically meaningful information using computational models [29]. The high-dimensional ophthalmic data are ingested into AI algorithms, and structural patterns are recognised that are related to disease progression and ocular abnormalities [30]. However, integration of multimodal datasets introduces challenges related to data standardisation, interoperability, and variability in acquisition protocols, which may affect model reliability [31].

Multimodal Diagnostic Systems

Multimodal diagnostic systems integrate data that is derived using various ophthalmic modalities, such as fundus imaging, optical coherence tomography, visual field testing, and ocular biometric parameters. The combination of these mixed datasets will enable the description of both anatomical and functional alterations that occur during ocular diseases in detail [30]. There is increased capacity of multimodal trained AI models in detecting the early signs of pathological changes in retinal tissues and optic nerve morphologies [32]. Structural imaging and biometric measurements may be valuable as the same can be comcomitantly analysed to enhance the diagnostic sensitivity of a disease such as diabetic retinopathy, glaucoma, and myopic degeneration [33]. However, the imaging aspect coupled with biometric pointers via computational processes will aid in expressing the ocular health situation in its entirety, and which diagnostic markers are disease-specific [34].

AI Clinical Decision Support

**Figure 2:**
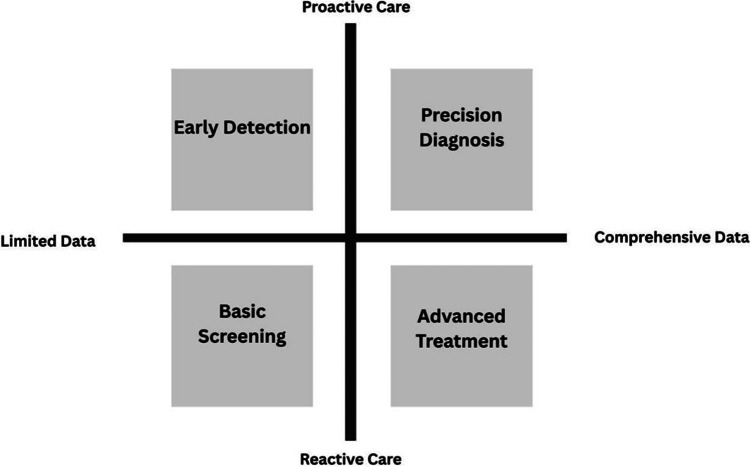
Data-Driven Framework for Proactive Ophthalmic Care

AI-based clinical decision support systems can provide ophthalmic diagnosis analysis and treatment plan analysis. These systems take into account the imaging, biometric data, and clinical features patterns to develop predictive models that can be utilised to explain complex ophthalmic data [35]. Automated analytic systems can rank the disease severity, assess the risk of disease progression, and recommend diagnostic classification using learned patterns based on annotated clinical data [36]. The use of computational decision support applications in digital ophthalmology systems can improve the efficiency of diagnostic and support evidence-based clinical management [37]. Despite these advantages, real-world implementation remains limited due to regulatory barriers, lack of large-scale prospective validation, and concerns regarding medico-legal accountability. The ever-increasing refinement of the algorithms of AI when using increasing volumes of ophthalmic data enhances the quality of the diagnosis and increases the applicability of the intelligent diagnostic systems to clinical practice in the ophthalmology field [38]. Figure 2 presents a conceptual diagram to depict how the transition to proactive ophthalmic care can be made by moving beyond the limited data integration to a comprehensive one.

Tele-optometry and remote ophthalmic screening

Tele-optometry is one of the most important innovations of the modern world in the field of providing eye care based on digital communication technologies, which help provide the remote services of the ocular examination and screening. The growth of telemedicine infrastructure has facilitated ophthalmic assessment outside of traditional clinical settings and has provided the opportunity to offer diagnostic services to geographically spread and underserved populations [3]. Electronic transfer of eye images and clinical data facilitates the coordination between primary care centres and ophthalmic institution centres [10]. The implementation of digital imaging systems in telemedicine systems has increased the screening of ocular conditions and enhanced continuity of ophthalmic services in the healthcare environment [13].

Remote Vision Assessment and Imaging

Remote vision screening will be based on portable diagnostic tools that have the potential to record high-quality eye images in non-specialised environments. Smartphone-based retinal imaging systems provide convenient and accessible platforms for capturing retinal structures and detecting pathological defects in community screening programs [33]. Smartphone cameras integrated with specialised optical attachments enable visualisation of the fundus and optic disc at a resolution sufficient for preliminary clinical assessment and remote consultation [33]. Portable ophthalmic cameras and handheld fundus cameras also increase the ability of remote retinal examination in primary care clinics, in rural health facilities, as well as outreach screening programmes [6]. Digital connectivity leads to the possibility of transmitting captured images safely to ophthalmologists or trained graders so that images can be diagnosed.

Image Assessment

Remote image assessment helps in the detection of retinal diseases such as diabetic retinopathy, hypertensive retinopathy, and macular degeneration as part of tele-ophthalmology [1]. Tele-retinal screening program implementation enhances the early image recognition of ocular disorders that pose risks to vision by conducting periodic check-ups for those at-risk groups [6]. These screening programs help in making referrals to specialised ophthalmic consultation promptly and enhance the effectiveness of diagnostic systems in healthcare systems [3]. In addition, the effectiveness of tele-optometry systems is contingent upon stable digital infrastructure and secure data transmission, which may not be consistently available in resource-limited settings.

Longitudinal observation of ocular conditions with the help of periodic digital imaging and remote follow-up assessment is also facilitated by tele-optometry platforms. The connection between portable imaging equipment and electronic health records and cloud storage systems promotes continuity of patient care and effective time-based disease progression tracking [35]. Screening tools that are supported by AI and installed in the tele-ophthalmology systems further enhance the efficiency of the diagnostic process as they allow automated interpretation of the retinal images and rank the patients that demand clinical care [18]. The ongoing advancement of remote ophthalmic screening devices enhances preventive eye-care measures and helps in managing vision health at the level of the entire population [31].

Clinical and public health impact of digital eye-care diagnostics

The digital ophthalmic diagnostics has brought quantifiable changes in disease identification, the effectiveness of mass screening, and the availability of vision care services. A combination of innovative imaging infrastructures, machine learning algorithms, and tele-optometry can allow ocular health evaluation on a large scale in various healthcare settings [29]. Digital diagnostic models assist in systematised screening of retinal and optic nerve diseases, enhancing detection of vision-threatening conditions at earlier points of clinical progression [30]. Early detection of pathological evidence of retinal tissue, optic nerve morphology, and anterior segment morphology facilitates early therapeutic intervention and limits the chances of a permanent loss in vision [31].

Early Disease Detection

AI algorithms to analyse retinal images can further improve the detection of the presence of subtle pathological features linked to diabetic retinopathy, glaucoma, and age-related macular degeneration [29]. Digital imaging modalities offer visualisation of the ocular structures with high precision, which permits detecting the abnormalities in microvascular elements, structural thinning, and initial degenerative alterations even earlier before the vision loss manifests itself [32]. Combining the use of computational diagnostic tools and retinal imaging data can lead to the objective interpretation of intricate ophthalmic data and the uniform screening results across healthcare systems [36]. The use of systematic screening programs with the help of digital diagnostic technologies enhances the detection of a high-risk group of people with diabetes, hypertension, and advanced age [31]. However, long-term evidence demonstrating improved clinical outcomes and cost-effectiveness remains limited, highlighting the need for longitudinal validation studies.

Accessibility and Healthcare Equity

Electronic ophthalmic diagnostic devices lead to increased available vision care services at the population level. Tele-optometry systems can be used to send ocular images and clinical records between the community health centre and the special ophthalmic clinic, as well as to provide a diagnostic consultation at a distance [37]. Portable retinal imaging equipment can be used to aid screening programs in rural areas, primary healthcare clinics, and outreach programs to minimise the obstacles related to geographical distance and scarcity of specialists [38]. Digital platforms based on the cloud enable safe management of ophthalmic datasets and multidisciplinary clinical team coordination of the evaluation [39]. This type of digital infrastructure facilitates fair allocation of ophthalmic care and enhances preventive eye-care measures among the underserved groups [40]. Major public health benefits associated with digital eye-care diagnostics are summarised in Table 3.

**Table 3:** Public Health Impact of Digital Ophthalmic Diagnostic Technologies Compiled by the authors from multiple sources [29-31,33,34]. AI: Artificial Intelligence, DR: Diabetic Retinopathy, OCT: Optical Coherence Tomography, AMD: Age-Related Macular Degeneration

Impact Area	Digital Technology Used	Public Health Benefit	Target Population	Implementation Setting	Supporting Reference
DR screening	AI-assisted fundus imaging	Early detection of retinal microvascular disease	Patients with diabetes	Community screening programs	[30]
Glaucoma detection	OCT with AI optic nerve analysis	Identification of early optic nerve damage	Adults at risk of glaucoma	Ophthalmology clinics	[31]
AMD monitoring	Retinal imaging with automated analysis	Monitoring macular degeneration progression	Elderly population	Tele-ophthalmology services	[29]
Vision screening	Portable retinal cameras	Expansion of diagnostic coverage	Rural and underserved populations	Primary care facilities	[33]
Population surveillance	Tele-optometry networks with AI analytics	Improved accessibility of eye-care services	Large population groups	Public health screening initiatives	[34]

Limitations and future recommendations

The use of AI, biometric testing, and tele-optometry in ophthalmic diagnostics remains constrained by a number of limitations. Most AI models are based on data obtained with narrow groups of demographics, making it less likely to generalise to different populations. Furthermore, many models lack robust external validation across heterogeneous datasets, reducing confidence in their generalizability to real-world clinical environments. The heterogeneity caused by variability in imaging protocols, device calibration, and standardisation of annotations can have an impact on the reliability of algorithms. Poor digital infrastructure, in particular in areas with limited resources, restricts the implementation of advanced imaging systems and tele-optometry platforms. Big data aspects of clinical integration of digital diagnostic technologies are also hampered by concerns of data governance, data privacy, and control of regulations.

Growing digital ophthalmic diagnostics require the expansion of multicentric datasets containing heterogeneous populations and imaging systems. Consistent imaging guidelines and data interoperable platforms may help increase algorithmic stability and diagnostic consistency. Multimodal datasets, such as imaging, biometric parameters, and clinical indicators, are likely to be better predictors of ocular diseases. Increased distribution of portable diagnostic equipment and tele-optometry networks can help improve access to vision screening in underserved areas. The concerted efforts of the clinicians, developers of the technologies, and the regulators can assist in the effective and safe deployment of digital ophthalmic diagnostic systems.

## Conclusions

Digitisation in ophthalmology has improved diagnostic accuracy, early disease detection, and access to vision care services. Integration of AI, ocular biometric analysis, and tele-optometry enables automated image interpretation, precise anatomical assessment, and remote screening. These technologies support early identification of conditions such as diabetic retinopathy, glaucoma, and age-related macular degeneration, while facilitating personalised clinical evaluation and expanding access to underserved populations. Continued advances in validation, standardisation, and infrastructure are required to enhance reliability and scalability. However, challenges related to data generalisability, regulatory implementation, and integration into clinical workflows remain important considerations. Overall, integrated digital ophthalmic diagnostics hold significant potential for improving population-level screening and global eye-care delivery. Nonetheless, challenges related to validation, standardization, and regulatory implementation must be addressed to ensure safe and effective clinical deployment.
